# Ultralow-density *Plasmodium falciparum* Infections in African Settings

**DOI:** 10.1093/cid/ciz147

**Published:** 2019-02-15

**Authors:** Shehu S Awandu, Jaishree Raman, Teun Bousema, Lyn-Marie Birkholtz

**Affiliations:** 1 Department of Biochemistry, Genetics and Microbiology, Institute for Sustainable Malaria Control & Medical Research Council Collaborating Centre for Malaria Research, University of Pretoria, South Africa; 2 Department of Medical Microbiology, Radboud University Medical Centre, Nijmegen, The Netherlands; 3 Centre for Emerging Zoonotic and Parasitic Diseases, National Institute for Communicable Diseases; 4 Wits Research Institute for Malaria Research, University of Witwatersrand, Johannesburg, South Africa

To the Editor—As countries accelerate towards elimination, an increasing proportion of infections may be of low parasite densities. In a recent report, Girma and colleagues [1] deployed ultrasensitive diagnostics to characterize asymptomatic infections in Ethiopia. The *Plasmodium falciparum* prevalence was 1.3% by microscopy, 3.6% by conventional rapid diagnostic tests (RDT), 8.5% by ultrasensitive Alere RDT, 22.2% by loop-mediated isothermal amplification and 21.5% by ultrasensitive quantitative reverse transcription-polymerase chain reaction (qRT-PCR). These findings are in line with a growing body of evidence demonstrating the superiority of ultrasensitive diagnostics in detecting low-density infections, when compared to microscopy and standard RDTs [2]. The reported qRT-PCR prevalence is considerably higher than prevalence estimates from a meta-analysis tool that relates microscopy and PCR prevalence data from population surveys [3]. Based on the meta-analysis, one would expect a *P. falciparum* PCR prevalence in the range of 2.9% to 10.6%. The higher prevalence in the study by Girma and colleagues [1] may be explained by their approach to targeting highly abundant RNA targets instead of DNA targets. Their finding thus suggests that there may be a reservoir of infections that is too low to be detected by conventional diagnostics or even conventional PCR [4]. Our own findings, from cross-sectional surveys in pre-elimination settings of South Africa, are in line with the findings of Girma and colleagues [1], in the sense that we also detected infections with ultralow parasite densities, below the limit of detection of conventional PCR. Our study observed no RDT-positive infections or 18S nested-PCR–positive infections among 1475 individuals, whilst 3.9% of the study population was positive for *P. falciparum* parasites by sensitive, telomere-associated repetitive element 2–based quantitative PCR (qPCR), sometimes with genetically complex infections (Table 1).

**Table 1.** *Plasmodium falciparum* Infection and Multiplicity of Infection Outcomes in South Africa

	Local Subjects	Migrant Subjects
RDTs (First Response Malaria)	0 (0/933)	0 (0/542)
18S rRNA PCR	0 (0/933)	0 (0/542)
TARE-2 qPCR % (n/N)	2.6% (24/993)	6.1% (33/542)
Mean multiplicity of infections (range)	1.8 (1–3)	2.8 (1–5)

Subjects were recruited in 2 community-wide, cross-sectional surveys among asymptomatic participants in 2014 and 2015. The 18S rRNA PCR [5], TARE-2 qPCR [6], and multiplicity of infections [7] were based on established protocols, using 4.2 µL of blood from filter paper bloodspots. Abbreviation: PCR, polymerase chain reaction; RDT, rapid diagnostic tests; rRNA, ribosomal RNA; TARE-2 qPCR, telomere-associated repetitive element-2 quantitative PCR.

The real challenge of the study by Girma and colleagues [1], as well as of our own work, lies in the interpretation of such parasite survey data in relation to transmission patterns, particularly in low-transmission settings. Ethiopia and South Africa have both set targets for malaria elimination. It is unclear to what extent the presence of ultralow-density infections may challenge these ambitions. The authors correctly point out the limitations of cross-sectional surveys for answering such questions, since these fail to take into account parasite dynamics that may fluctuate on a daily basis [8]. The authors also did not perform any assessment of gametocyte carriage or transmissibility to mosquitoes, whilst longitudinal surveys that accurately measure parasite kinetics, gametocyte production, and onward transmission potential are probably needed to truly determine the relevance of low-density infections for onward transmission. This contribution to transmission not only depends on their infectivity to mosquitoes, but also on real-life mosquito exposure [9]. In areas with low vector densities, inefficient vectors, or effective vector control, the transmission potential of low-density or ultralow-density infections is likely to be very limited. In other settings, such infections may plausibly form a stumbling block for elimination [10]. The study by Girma and colleagues [1] thereby forms a relevant starting point to examine these important questions, which urgently need addressing to inform malaria policy.

## Note

***Potential conflicts of interest.***All authors: No reported conflicts. All authors have submitted the ICMJE Form for Disclosure of Potential Conflicts of Interest. Conflicts that the editors consider relevant to the content of the manuscript have been disclosed.
